# Genetic and geographical delineation of zoonotic vector-borne helminths of canids

**DOI:** 10.1038/s41598-022-10553-w

**Published:** 2022-04-24

**Authors:** Younes Laidoudi, Samia Bedjaoui, Maria Stefania Latrofa, Angela Fanelli, Filipe Dantas-Torres, Domenico Otranto

**Affiliations:** 1 Parasitology Unit, Department of Veterinary Medicine, University of Bari, Valenzano, Italy; 2 PADESCA Laboratory, Veterinary Science Institute, University of Constantine 1, 25100 El Khroub, Algeria; 3 Laboratory of Food Hygiene and Quality Insurance System (HASAQ), Higher National Veterinary School, Issad Abbes, Oued Smar, 16000 Algiers, Algeria; 4 Infectious Diseases Unit, Department of Veterinary Medicine, University of Bari, Valenzano, Italy; 5 Laboratory of Immunoparasitology, Department of Immunology, Aggeu Magalhães Institute, Oswaldo Cruz Foundation (Fiocruz), Recife, Pernambuco 50740-465 Brazil; 6 Faculty of Veterinary Sciences, Bu-Ali Sina University, Hamedan, Iran

**Keywords:** Genetics, Genotype, Haplotypes, Population genetics

## Abstract

Several zoonotic vector-borne helminths (VBHs) infesting canids cause serious veterinary and medical diseases worldwide. Increasing the knowledge about their genetic structures is pivotal to identify them and therefore to settle effective surveillance and control measures. To overcome the limitation due to the heterogeneity of large DNA sequence-datasets used for their genetic characterization, available cytochrome *c* oxidase subunit 1 (*cox*1) (n = 546) and the 12S rRNA (n = 280) sequences were examined using combined bioinformatic approach (i.e., distance-clustering, maximum likelihood phylogeny and phylogenetic evolutionary placement). Out of the 826 DNA available sequences from GenBank, 94.7% were characterized at the haplotype level regardless sequence size, completeness and/or their position. A total of 89 different haplotypes were delineated either by *cox*1 (n = 35), 12S rRNA (n = 21) or by both genes (n = 33), for 14 VBHs (e.g., *Acanthocheilonema reconditum*, *Brugia* spp., *Dirofilaria immitis*, *Dirofilaria repens*, *Onchocerca lupi* and *Thelazia* spp.). Overall, the present approach could be useful for studying global genetic diversity and phylogeography of VBHs. However, as barcoding sequences were restricted to two mitochondrial loci (*cox*1 and 12S rRNA), the haplotype delineation proposed herein should be confirmed by the characterization of other nuclear loci also to overcome potential limitations caused by the heteroplasmy phenomenon within the mitogenome of VBHs.

## Introduction

Zoonotic vector-borne helminths (VBHs) of canids include a cosmopolitan group of heteroxenous parasitic worms belonging to Onchocercidae and Thelaziidae families^1–3^ (Table 1). Adult helminths colonise tissues and body cavities of the vertebrate hosts and produce blood-, skin- or even mucus-dwelling L1 larvae, that are ingested by arthropod vectors^4,5^ in which they undergo through two developmental stages, until the infective form (L3)^6^. Canids, especially dogs, are the suitable hosts for several zoonotic onchocercids (i.e., *Dirofilaria repens*, *Dirofilaria immitis*, *Brugia* spp., and *Onchocerca lupi*) and thelaziid parasites (i.e., *Thelazia callipaeda* and *Thelazia californiensis*)^7–9^. Some of VBHs (i.e., *Dirofilaria* spp. and *O. lupi*) are of growing concern due to their spread to new regions^10,11^, zoonotic significance, and associated morbidity in animals and humans^4,5^. Expanding the knowledge on their biology, ecology and geographical distribution constitutes a key point for planning effective surveillance and control measures. In this context, the study of specific gene targets (e.g., DNA barcode) has represented an advancement in the understanding of the taxonomy, molecular epidemiology, and population genetic of this group of parasites^12^. However, except for the few studies on the population genetic of *T. callipaeda*, *D. repens* and *O. lupi*^13–16^, most studies involving DNA-based barcoding are focused on species identification. For such purposes, the characterization of a single DNA marker often suffices, resulting in the arbitrary use of numerous PCR assays by research and diagnostic laboratories which led to the creation of heterogeneous gene databases of non-universalised target genes and amplicons. Overall, these non-homogeneous data may constitute the first barrier toward the knowledge on population genetic structure, phylogeography and biology of these parasites.

**Table 1:** Supplementary information of zoonotic VBH of canids^4–6,16^.

Subfamilies	Species and authority name	Family of parasitized hosts	Anatomical infection site	Genera of vectors	Geographic distribution
Dirofilariinae	*Dirofilaria* (*Dirofilaria*) *immitis* (Leidy, 1856)	Canidae, Felidae, Otariidae, Mustelidae, Ursidae, Castoridae, Procyonidae, Leporidae, Cervidae, Equidae, Hominidae, Microtidae	Heart	*Aedes, Anopheles, Mansonia, Culex*	Europe, Asia, Africa
	*Dirofilaria* (*Nochtiella*) *repens* Railliet & Henry, 1911	Canidae, Felidae	Subcutaneous connective tissues	*Aedes, Anopheles, Mansonia, Culex*	Cosmopolitan
	*Dirofilaria tenuis* (Chandler, 1942)	Procyonidae	Subcutaneous tissues	*Aedes, Anopheles, Psorophora*	USA
	*Dirofilaria ursi* (Yamaguti, 1941)	Ursidae	Subcutaneous tissues	*Simulium*	Japan, North America and Russia
	*Dirofilaria striata* (Molin, 1858)	Canidae, Felidae	Subcutaneous connective tissues and intermuscular fascia	*Aedes, Anopheles, Culex*	North and South America
Splendidofilariinae	*Acanthocheilonema reconditum* (Grassi, 1889) (syn. *Dipetalonema reconditum*)	Canidae	Subcutaneous tissues and fascia	*Ctenocephalides, Heterodoxus, Linognathus*	Cosmopolitan
Onchocercinea	*Onchocerca lupi* (Rodonaja, 1967)	Canidae	Connective tissue of the sclera, Ocular	*ND*	North America, Europe, Asia
	*Brugia malayi* (Brug, 1927)	Canidae, Hominidae, Felidae, Viverridae, Manidae	Lymphatic vessel and ganglions	*Mansonia, Anopheles, Aedes*	Eurasia, North Africa
	*Brugia pahangi* (Buckley & Edeson, 1956)	Canidae, Cebidae, Erinaceidae, Felidae, Lorisidae, Manidae, Scuiridae, Viverridae	Lymphatic vessel and ganglions	*Mansonia, Anopheles, Armigeres, Psorophora, Culex, Aedes*	Asia
	*Brugia ceylonensis* (Jayewardene, 1962)	Canidae	Lymphatic vessel and ganglions	*Aedes, Anopheles, Mansonoides*	Sri Lanka
	*Brugia patei* (Buckley, Nelson and Heisch, 1958)	Canidae, Felidae	Heart and pulmonary artery, testes, and associated lymphatics	*Mansonia, Aedes*	Kenya
Thelaziinae	*Thelazia californiensis* (Price, 1930)	Canidae, Felidae	Eye	*Fannia*	Western North America
	*Thelazia callipaeda* (Railliet and Henry, 1910)	Canidae, Leporidae, Felidae	Eye	*Phortica*	The CIS, Europe, China, Japan, India, Burma, and Korea

Parasite subfamilies, parasitized host species, anatomical infection sites, vector genera and geographic distribution are provided.

CIS: Commonwealth of Independent States which includes Armenia, Azerbaijan, Belarus, Georgia, Kazakhstan, Kyrgyzstan, Moldova, Russia, Tajikistan, Turkmenistan, Ukraine, and Uzbekistan. ND: not determined.

A notable increase in the number of softwares and algorithms lead to more refined use of molecular information in taxonomic works^12^. Most common tools and algorithms for the assessment of genetic diversity and species delimitation were based either on a phenetic (e.g., ASAP and ABGD)^17,18^ or on phylogenetic (e.g., (m)GMYC and (m)PTP) criteria, requiring a well-defined DNA fragment from a multisequence alignment as a starting template^19–22^. However, the usefulness of these tools was limited by the heterogenous datasets (i.e., differences in sequence length and localisation), making the characterization based on these DNA gene targets less adapted, also considering that the genetic and evolutionary relationships differ among and between species for a given barcode gene^23^. Moreover, the use of more than one DNA-barcode marker is advocated for species delineation and taxonomic works^18^.

In the last 20 years, genetic studies on VBHs have mainly focused on the characterization of two mitochondrial loci (i.e., the 12S rRNA and cytochrome *c* oxidase subunit 1, *cox*1), which provided new and refined information on the biology and epidemiology of VBHs. In addition, some epidemiological and molecular studies based on mitochondrial DNA barcoding markers attempted to unearth the genetic diversity of some of these species (e.g., *T. callipaeda*, *O. lupi* and *D. repens*)^13,14^. However, these studies were either focused on phylogenies for assessing the genetic diversity of a limited number of species^13,14^ or simple genetic identification, all involving a limited number of representative sequences. Therefore, a comprehensive metabarcoding approach is timely to study the zoonotic VBHs globally. Aware of the limited quality and provenance of the DNA sequences available in the public database (GenBank), we used a combined approach based on genetic distance clustering, maximum likelihood (ML) phylogeny and evolutionary placement algorithm to describe the genetic diversity and create a comprehensive platform for monitoring the diversity and phylogeography of these parasitic nematodes.

## Results

Overall, 546 and 280 sequences, representing A and B datasets respectively, were available from GenBank database for the zoonotic VBHs, which covers 47 countries and 18 vertebrate hosts other than dogs and cats and humans (Supplemental Table S1). These datasets lack DNA sequences from the zoonotic *Dirofilaria tenuis*, *Brugia ceylonensis* and *Brugia patei*. The core sequence alignment (CoSA) of 652 and 413 bp was identified through the A and B datasets. Sequence clustering analysis yielded the delineation of 68 and 54 reference sequences from A and B datasets respectively. Of those, 33 reference sequences were mapped from both datasets and were used as dataset C.

Overall, 89 different haplotypes identified for *cox*1 (n = 35), 12S rRNA (n = 21) or for both genes (n = 33), representing the characterization of 14 species (11 described and three referred to as at the genus level) (Supplemental Table S1). Similarly, the evolutionary phylogenetic placement obtained by the EPA-ng algorithm reproduced the same haplotype delineation of sequences from A, B and C datasets with a weight ratio ranging between 0.85 and 1.

**Figure 1:**
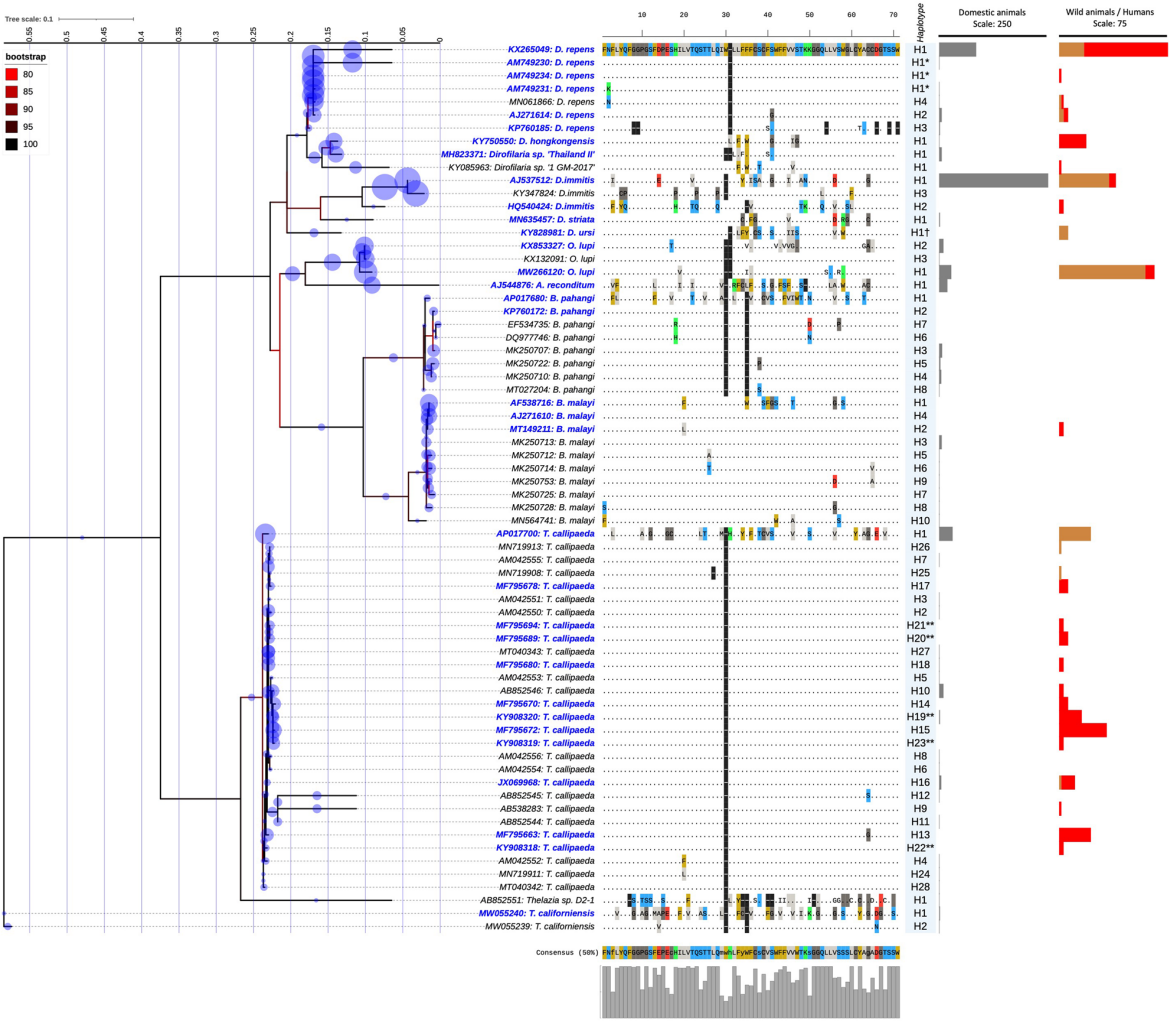
ML phylogeny showing the distribution of the haplotypes of zoonotic VBHs of canids delineated by the *cox*1 distance clustering. The tree corresponds to the IQTREE inferred from 68 partial (651 bp) DNA sequences with 32.7% of informative sites using the TIM3 (+F+I+G4) model under 1000 bootstrap replicates and ML method. Branch are color-coded according to the bootstrap value. Species name and GenBank accession number are indicated at the tip of each branch. Bold blue label indicated the reference sequences used in the MLST phylogeny. The tree includes 478 query sequences from the zoonotic VBHs of canids (blue circle) placed at the branch and leaf nodes by the EPA-ng algorithm. The MY_SCHEME_1 sequence alignment viewer of the informative sites from the amino acid alignment and their 50% consensus are shown. The amino acid sequences of the haplotype 1 from each species were used as reference sequence to dot repeats in amino acids throughout the whole haplotypes of the species. Haplotype names are indicated for each node label. Number of domestic, wild, and human infection cases with each haplotype are shown by the bar charts at each node. * and ** indicate delineation failure of the *cox*1 and 12S rRNA distance clustering respectively. ^†^ Indicates inconsistency between the morphological taxonomy and the present molecular characterization.

**Figure 2:**
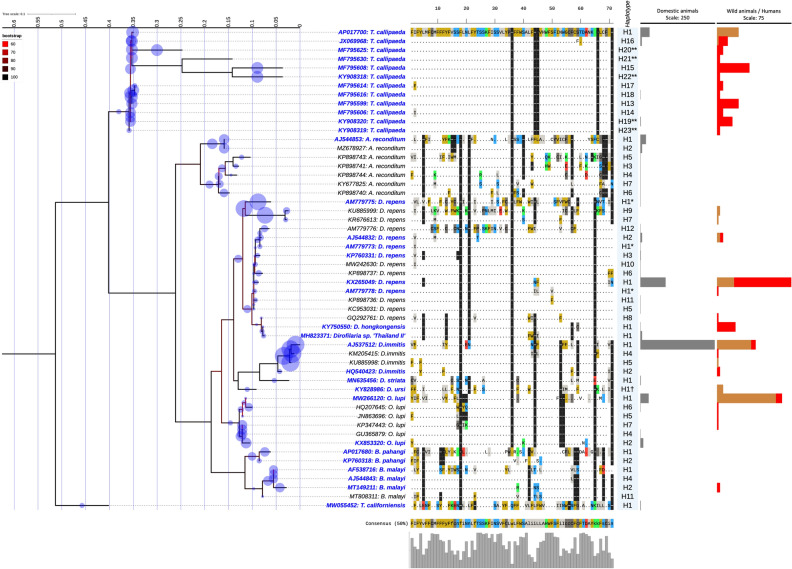
ML phylogeny showing the distribution of the haplotypes of zoonotic VBHs of canids delineated by the 12S rRNA distance clustering. The tree corresponds to the IQTREE inferred from 54 partial (413 bp) DNA sequences with 48.9% of informative sites using the TIM2 (+F+R4) model under 1000 bootstrap replicates and ML method. Branch are color-coded according to the bootstrap value. Species name and GenBank accession number are indicated at the tip of each branch. Bold blue label indicated the reference sequences used in the MLST phylogeny. The tree includes 226 query sequences from the zoonotic VBHs of canids (blue circle) placed at the branch and leaf nodes by the EPA-ng algorithm. The MY_SCHEME_1 sequence alignment viewer of the informative sites from the amino acid alignment and their 50% consensus are shown. The amino acid sequences of the haplotype 1 from each species were used as reference sequence to dot repeats in amino acids throughout the whole haplotypes of the species. Haplotype names are indicated for each node label. Number of domestic, wild, and human infection cases with each haplotype are shown by the bar charts at each node. * and ** indicate delineation failure of the *cox*1 and 12S distance clustering respectively. ^†^ Indicates inconsistency between the morphological taxonomy and the present molecular characterization.

**Figure 3:**
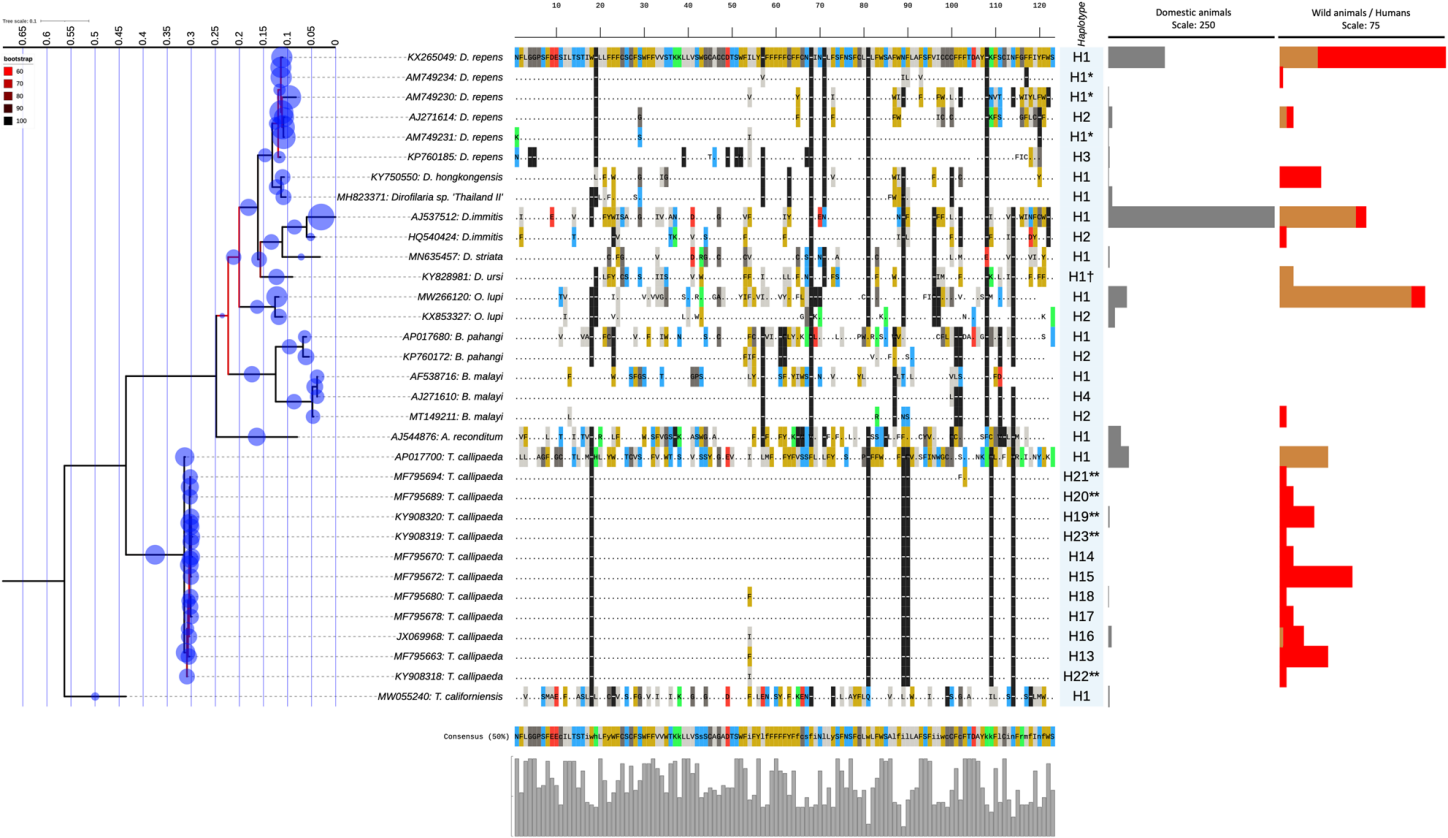
ML phylogeny showing the distribution of the haplotypes of zoonotic VBHs of canids delineated by the *cox1* and 12S rRNA distance clustering. The tree corresponds to the IQTREE inferred from 33 concatenated (1064 bp) DNA sequences with 29.13% of informative sites using the (+F+R5) model under 1000 bootstrap replicates and ML method. Branch are color-coded according to the bootstrap value. Species name and GenBank accession number are indicated at the tip of each branch. The tree includes 704 (478 *cox*1 and 226 12S rRNA) partial query sequences from the zoonotic VBHs of canids (blue circle) placed at the branch and leaf nodes by the EPA-ng algorithm. The MY_SCHEME_1sequence alignment viewer of the concatenated informative sites from the cox1 and 12S amino acid alignment and their 50% consensus are shown. The amino acid sequences of the haplotype 1 from each species were used as reference sequence to dot repeats in amino acids throughout the whole haplotypes of the species. Haplotype names are indicated for each node label. Number of domestic, wild, and human infection cases with each haplotype are shown by the bar charts at each node. * and ** indicate delineation failure of the *cox*1 and 12S rRNA distance clustering respectively. † Indicates inconsistency between the morphological taxonomy and the present molecular characterization.

Single and multilocus analyses combining sequence clustering, ML phylogeny and the EPA-ng placement yielded the identification of 94.7% (782/826) and 81.1% (699/826) sequences from all species at the haplotypes level respectively (Figs. 1, 2, 3). However, 6.9% (38/546) and 2.1% (6/280) of sequences from A and B datasets, respectively, were considered as putative haplotypes based on distance clustering and the EPA-ng placement (like weight ratio < 0.85) and the limited query cover on the CoSA. These sequences were of *T. callipaeda* (two *cox*1), *O. lupi* (two 12S rRNA), *A. reconditum* (one *cox*1 and three 12S rRNA), *D. repens* (33 *cox*1 and one 12S rRNA) and from *Dirofilaria* sp. subgenus *Nochtiella* (*cox*1 accession number: GU474429) (Supplemental Table S1).

Delineation failure was observed for three *D. repens* haplotypes, where the 12S rRNA gene yielded their discrimination, whilst the *cox*1 sequences were haplotype 1. Conversely, the *cox*1 clustering revealed a discriminatory delineation of five *T. callipaeda* haplotypes (e.g., 16, 19, 20, 21 and 23) having a similar 12S rRNA sequences (Supplemental Table S1). For *T. callipaeda*, synonymous mutations were most frequent in both genes (Figs. 1, 2, 3), whilst for the quested Onchocercidae species, most DNA mutations in both loci were non-silent and induced a change in the protein's amino-acid sequence of all delineated haplotypes (Figs. 1, 2, 3). The analysis delineated seven new haplotypes (H22-28) for *T. callipaeda* from humans, pets and wild animals from China (Supplemental Table S1). Two newly haplotypes of *T. californiensis* were herein delineated by the *cox*1 gene. These sequences (*cox*1 accession number: MW055239-40) were from male and female worms isolated simultaneously from the same dog^24^. Sequence comparison revealed the presence of up to six transitions (i.e., three A>G, two T>C and one C>T) and one transversion (i.e., T>A). Of these, two transitions (A>G) induced a change in protein sequence (i.e., Alanine to Valine and Aspartic acid to Aspargin) (Fig. 1). Twelve haplotypes of *D. repens* were delineated for both genes (H1-3), one by the *cox*1 (H4) or by the 12S rRNA (H5-12), followed by *B. malayi* with 11 haplotypes, *B. pahangi* eight haplotypes, *A. reconditum* and *O. lupi* with seven for each and five haplotypes for *D. immitis* delineated by *cox*1 (n = 1), 12S rRNA (n = 2) or by both genes (n = 2). Overall, a less genetic diversity was observed for the remaining species/subspecies (Supplemental Table S1).

**Figure 4:**
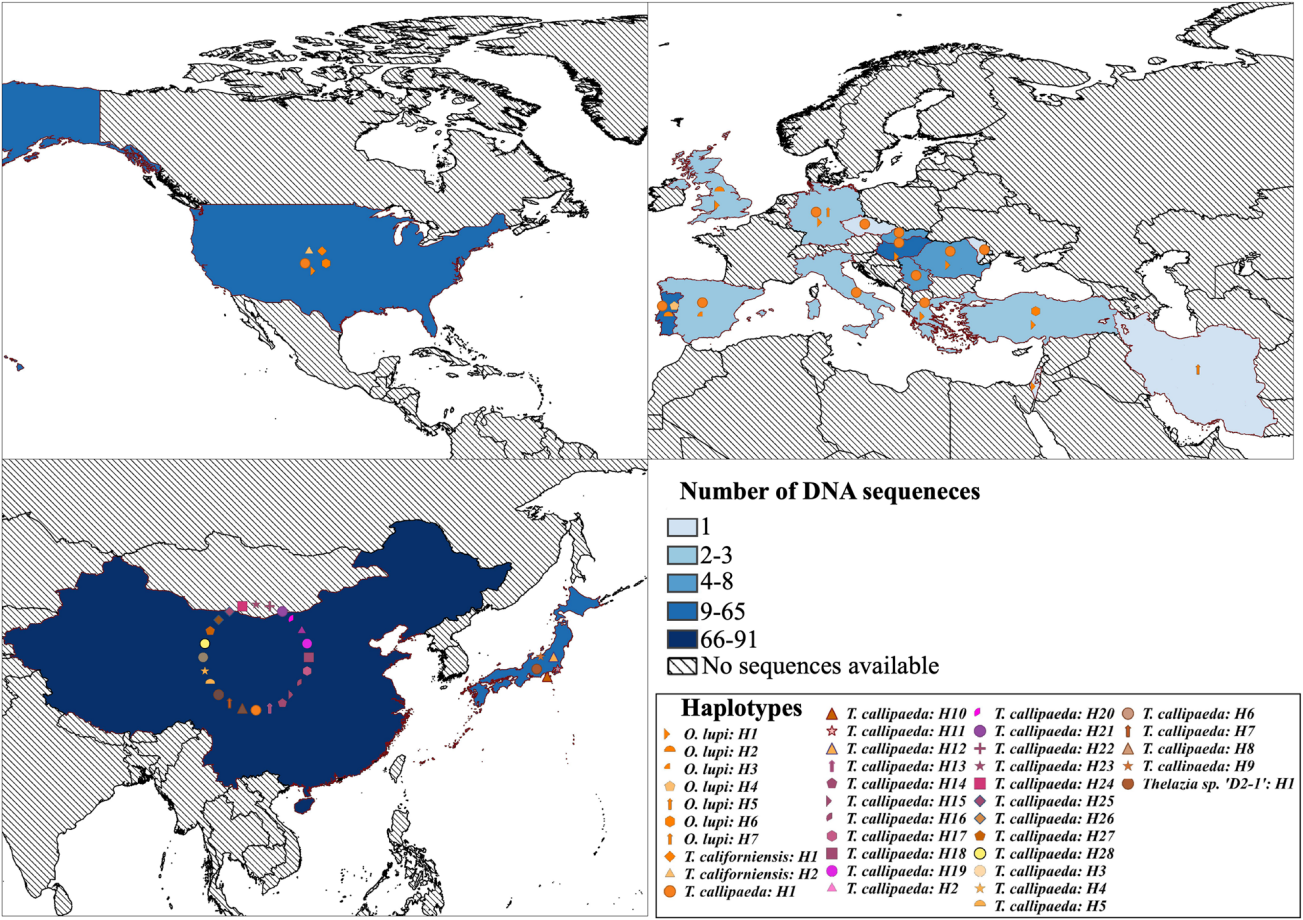
Geographical plotting of the zoonotic eye-worms (*Thelazia* spp. and *O. lupi*) haplotypes using the QGIS software (version 3.0.0, http://qgis.osgeo.org)^25^. The choropleth map (color-gradient map) represents the availability of DNA sequences per each country. Haplotypes for each species are plotted using country centroids and the point displacement tool.

**Figure 5:**
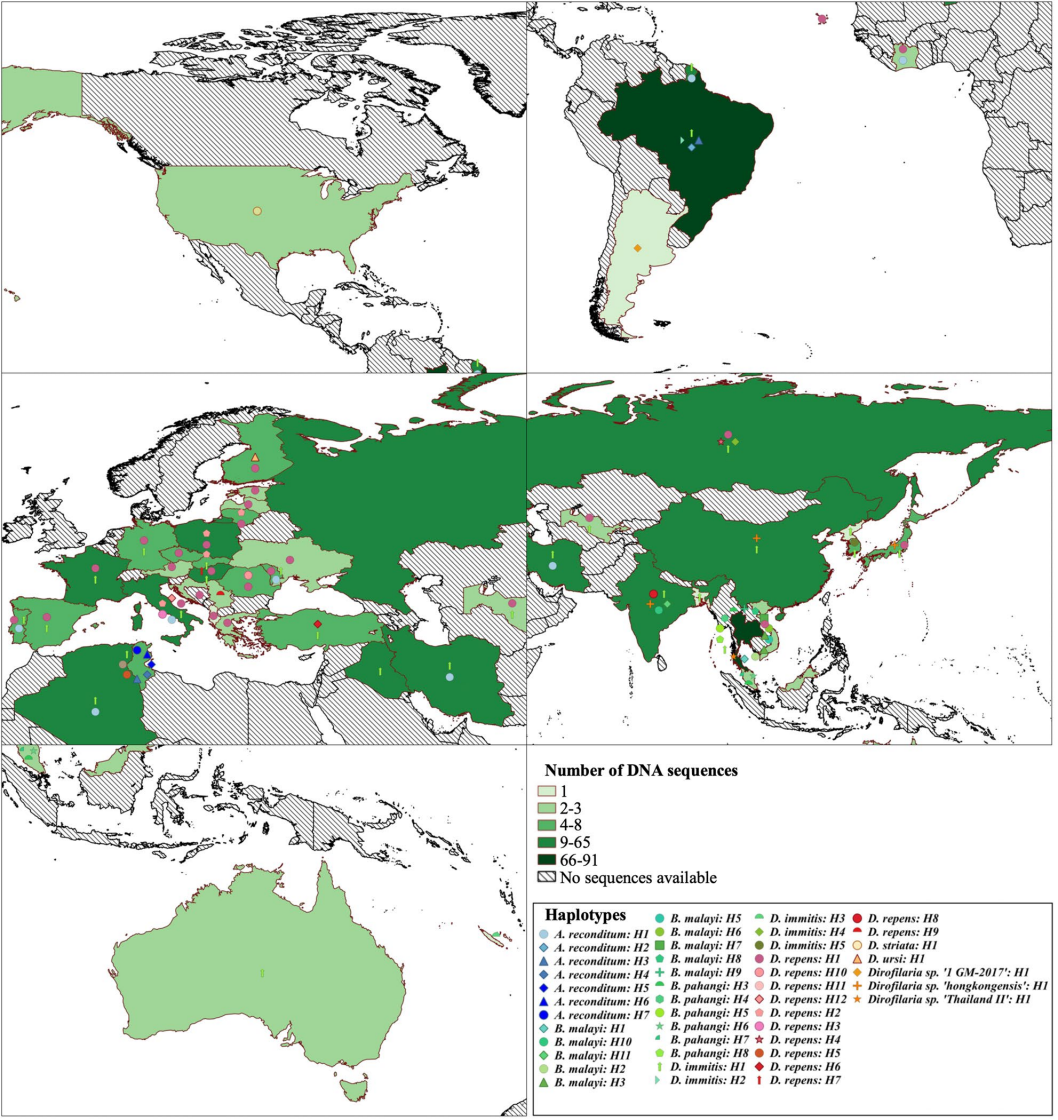
Geographical plotting of the zoonotic onchocercid haplotypes using the QGIS software (version 3.0.0, http://qgis.osgeo.org)^25^. The choropleth map (color-gradient map) represents the availability of DNA sequences per each country. Haplotypes for each species are plotted using country centroids and the point displacement tool.

Regarding the epidemiological importance, haplotype 1 of *D. immitis*, *D. repens*, *O. lupi* and *T. callipaeda* was the most frequently detected and geographically distributed one (Figs. 4, 5 and Supplemental Fig. S1). Except from *T. callipaeda*, these haplotypes were the most frequently involved in human cases (Figs. 1, 2, 3 and Supplemental Table S1).

## Discussion

The present study delineated the inter- and intraspecies diversity of selected VBHs infesting canids, based on the following criteria: (i) a core sequence alignment covering the most identified informative regions of barcoded specimens, (ii) distance clustering with predetermined threshold adjusted to each gene, (iii) the ML phylogeny inferred from the CoSA of reference sequences, and (iv) the phylogenetic evolutionary placement of query sequences with high-like weight ratio. Combining all these criteria, 94.7% of DNA sequences from datasets A and B were characterized at the genotype level regardless the sequence size or region, suggesting that this new approach could be useful for studying global genetic diversity from large DNA databases. Despite the exhaustive BLAST search herein performed, DNA loci from *D. tenuis*, *B. ceylonensis* and *B. patei* remains unavailable. These species need several clarifications. For example, a human dirofilariasis infection causing neuritis was confined to *D. tenuis* in South Florida (USA) without being confirmed by molecular data^26^. Moreover, a suspected *B. ceylonensis* involved in a human filariasis in Sri Lanka was previously reported^27^. It should also be noted that *B. ceylonensis* and *B. patei* are not completely resolved morphologically^6^, which may explain the absence of the confirmation of the human case from Sri Lanka^27^. In addition, some species such as *Thelazia* sp. (GenBank accession number: AB852551), and *Dirofilaria* spp. (GenBank accession numbers: KY085963, GU474429 and MH823371) were herein resolved at the genus level. However, the question arises whether they are new species cannot be ruled out in the absence of morphological data. Given that, further studies combining molecular and morphological characterization are needed to assess the identity/validity of these species. Although only a limited number of DNA loci were examined here, which could be a limitation of the present study, the delineation of haplotypes using the newly proposed approach was consistent with the previous description of *T. callipaeda* based on partial single mitochondrial loci (*cox*1)^28^ and on deep population genetic structure analysis^13^. In addition to the previously described haplotypes of *T. callipaeda* (H1-21)^13^, in this study seven new haplotypes were delineated from China (H22-28), thus expanding the genetic diversity of the parasite in this country. Interestingly, the occurrence of *T. callipaeda* infections in humans seems to be related to the phylogeography, with up to 74 DNA sequences representing 13 different Asian haplotypes of *T. callipaeda* reported from humans in Japan and China, while the haplotype 1 is the most diagnosed in domestic and wild animals from different geographical areas, especially from Europe. However, due to the absence of DNA sequences from human European cases of *T. callipaeda* reported so far, it remains to be ascertained whether the haplotype 1 is also linked to human cases. Nevertheless, considering the inconspicuous genetic differentiation of *T. callipaeda* from different animal species (i.e., dogs, foxes, cats, and humans), a lack of host specificity has been proposed in previous studies^29^. Moreover, the hypothesis linking the genetic diversity of *T. callipaeda* to the vector^13^ needs further confirmation.

In addition to the readily distinguishable putative species of the genus *Thelazia* from a Japanese dog (*Thelazia* sp. D2-1, GenBank accession number: AB852551), two other distinct haplotypes of zoonotic *T. californiensis* were described in the present study, expanding our knowledge on the species diversity. These two newly haplotypes of *T. californiensis* were from male and female worms isolated simultaneously from the same dog in New Mexico, USA^24^. Similarly, two *T. callipaeda* haplotypes were also detected in a single human patient in China^13^ likely due to the exposure to multiple haplotypes circulating in the infected drosophilid flies^13^. However, because of the limited number of *T. californiensis* sequences available*,* it is questionable if this diversity is based on the sympatric occurrence of different *T. californiensis* haplotypes in the USA or on the occurrence of heteroplasmy among *Thelazia* species, but this hypothesis needs to be proven. Heteroplasmy is a common condition of the coexistence of mutant and wild-type forms of mitochondrial DNA in the same cell of nematode worms^30,31^.

Five different haplotypes of *D. immitis* were detected, with the haplotype 1 being the most common worldwide, and haplotype 2 described causing human ocular dirofilariasis in South America^32,33^. The three other haplotypes have been described by only one DNA sequence each, obtained during routine epidemiological monitoring^34–36^. The presence of genetic diversity within *D. immitis* was confirmed by previous results based on the complete NADH dehydrogenase 1 (ND1) and 16S rRNA gene sequences of isolates from China, which highlighted two distinct phylotypes^37^. However, confirmation of similarity between the haplotypes described here and those from Liu et al. study cannot be ruled out because the target DNA sequences used in the two studies are different. This highlights the importance of universalizing the target DNA sequence for taxonomic and epidemiological purposes to strengthen the phylogeographic information. Unfortunately, sequence datasets assessed here lack the sequence from several morphologically valid species of the genus *Dirofilaria*^33^. In the same way, only one genotype was delineated for *D. ursi* and another one for *D. striata*, probably due to the paucity of DNA data available for these species. Interestingly, *D. ursi* clustered phylogenetically with the subgenus *Dirofilaria*, whilst this species was morphologically considered to be a part of the subgenus *Nochtiella*^38^. Thereby, a revision of the genus *Dirofilaria* under the framework of integrative taxonomy combining both morphological and molecular characters is needed.

At least 16 different haplotypes were retrieved in *D. repens-*like filarioids. The present study confirmed previous results based on the 2.5 kb mitochondrial fragment, containing the protein-coding genes for the NADH-ubiquinone oxidoreductase chain 1 (*nduo1*) and chain 4 (*ndfl4*), the small rRNA gene and the highly variable AT-rich non-coding control region of the *D. repens-*like filarioid mitogenome^14^. Furthermore, the genetic diversity of *D. repens-*like filarioids appear to be linked to either geography or vertebrate host. For example, 12 haplotype of *D. repens* have been described in European countries, but only one *D. repens* haplotype (haplotype 9, GenBank accession number: GQ292761) was isolated from a man in India^39^. The geographical origin of this case was linked to southern India or Sri Lanka (India), because of the travel history of the patient^39^. However, due to the absence of any other data on *D. repens* haplotype 1 from this area and the European origin of the patient (Germany), the European origin of this haplotype of *D. repens* remains the most probable origin, suggesting a geo-related phylogeny of this species. In addition, haplotype 1 of *D. repens* was most widespread but geographically restricted to European countries, with exception of four cases from Asia. Of these, two were detected from humans after travel to Europe^40^, one from a jackal in Uzbekistan^41^, and one from a Vietnamese patient^42^. These features reinforce the specific phylogeography of *D. repens* and demonstrate the utility of genetic characterization in tracing the origin of the parasite, especially when the patient’s travel history is unknown^43^. In addition to *D. repens*, the diversity of *D. repens-*like filarioids includes *Dirofilaria* sp. Thailand genotypes^14^, a species previously referred to as “*Candidatus* Dirofilaria hongkongensis” (unavailable name)^44^; detected in Hong Kong and parts of India, as well as an undescribed species detected in Argentina^45^. While all these sequences were phylogenetically clustered within the “*hongkongensis*” clade, the genetic profile of these *D. repens-*like filarioids was remarkably linked to the vertebrate host, as suggested previously^14^. The genotype of *Dirofilaria* sp. Thailand and the undescribed *Dirofilaria* (*Nochtiella*) sp. from Argentina have only been detected in cats and dogs, respectively^14^. On the other hand, despite the analysis of all available DNA sequences, the genetic diversity revealed here was not exhaustive and the *D. repens*-like filarioids seem to be more diverse. This hypothesis could be supported by the presence of a *cox*1 sequence of an undescribed *D. repens*-like filarioid (*Dirofilaria* sp. MK-2010, GenBank accession number: GU474429) derived from a human-testicular filariasis case in Austria. This DNA sequence was considered here as putative haplotype due to the shortness of the DNA sequence, but phylogenetically clustered as a distinct genotype within the “*hongkongensis*” clade (data not shown). In addition, a remarkably high prevalence of *D. repens*-like microfilariae was reported in Giemsa-stained blood smears from cats in Selangor State, (Malaysia)^46^. These features highlight the need for more comprehensive and accurate genetic data to fully characterise the genetic profile and taxonomic identity of these filaroids as mentioned above.

A remarkable diversity with seven genotypes was recorded among sequences from the neglected zoonotic *O. lupi*. The number of haplotypes of *O. lupi* was previously investigated by Rojas et al. using three mitochondrial markers (*cox1*, 12S rRNA and NADH-ubiquinone oxidoreductase chain 5 (*nad5*)) and two phylogenetic methods (e. g., ML phylogeny and haplotype network)^47^. Rojas et al. reported two well-separated genotypes, one with worms from the Old and New Worlds and one from Portugal and Spain. However, due to the shortness of the 12S rRNA (298 bp) and *nad5* (393 bp) they used, only the *cox1* delimitation is discussed here. As an example, *O. lupi* reported as genotype 1^47^ was herein found to encompass four groups of haplotypes: (i) sequences from USA, Hungary, Israel, Greece and Germany, (haplotype 1), (ii) sequences from Portugal (haplotype 2), (iii) sequences from Spain (haplotype 3) and (iv) sequences from Turkey (haplotype 6). In addition, to the different methods used to delineate the phyletic diversity and the difference in the size of the analysed sequences (570 bp *versus* 652 bp in the present study), the sequence dataset of Rojas et al. lacks sequences from the other haplotypes delineated here, such as sequence from Germany (GenBank accession number: KP347443, haplotype 7), Portugal (GenBank accession number: GU365879, haplotype 4) and Iran (GenBank accession number: JN863696, haplotype 5), which explains the differences in haplotype delimitation.

Similarly, the results of this study have revealed a broad phyletic diversity of *A. reconditum*, a neglected but potentially zoonotic VBH of canids^48^. The haplotype 1 was the most frequently detected and widespread, while the other haplotypes were restricted to two geographical areas (haplotype 2 in Brazil and haplotypes 3 to 7 in Tunisia) and were not linked to any scientific publication. Considering the limited information available on these sequences, this genetic diversity could be explained by the possible impact of the different epidemiological contexts in these areas and/or by the diversity of the vector species involved. Nowadays, at least two vector species (i.e., the cat fleas, *Ctenocephalides felis*, and the chewing louse of dogs, *Heterodoxus spiniger*) are known to transmit *A. reconditum* from and between dogs during feeding, although other flea and louse species may be involved^49^. Therefore, further studies should be carried out to explore the biology, population genetic, ecology and epidemiology of this neglected parasite.

Finally, high haplotypic diversity has been described here for Brugian parasites infecting canids. In addition to the 11 and 12 haplotypes of the lymphatic filariasis agents *B. malayi* and *B. pahangi* from Asian countries, the presence of a putative new species (*Brugia* sp. CMT1) from French Guiana was also detected. This species has been described molecularly from primates^50^ and dogs^51^. On the other hand, molecular characterization of *Brugia* spp. is often performed using the 5S gene^52^, leading to limited molecular information from the quested gene datasets. In addition, *Brugia* spp. found in the Americas were thought to be more zoonotic than the classic Asian species^53^. For this reason, previous studies have highlighted the prevalence of other species in wild and domestic canids, such as *Brugia* sp. from the ring-tailed coatis (*Nasua nasua nasua*) and domestic dogs in Brazil^54^ and *Brugia guyanensis* from the lymphatic system of the coatimundi (*Nasua nasua vittata*) in the independent nation of Guyana^55^. Further integrative studies combining morphological and molecular data are needed for a proper characterization of *Brugia* spp. circulating in the Americas.

Increasing knowledge on the phylogeography of zoonotic VBHs is crucial for effective surveillance and control measures. Here, we proposed a bioinformatic approach for accurate massive characterisation of gene datasets. However, the success of DNA barcoding depends on how much is available for barcoded specimens and how efficient the information provided by the target DNA sequence is for phyletic diversity, genetic variation, and life history information^12^. Thereby, the present delineation based only on mitochondrial loci stills limited by the possible occurrence of heteroplasmy within the mitogenome of nematodes, a possibility little investigated in the VBHs included herein.

## Methods

### Sequence datasets

An exhaustive BLASTn search was performed to retrieve the mitochondrial loci (12S rRNA and *cox*1) of the zoonotic VBHs of canids (i.e., *Brugia* spp., *Dirofilaria* spp., *A. reconditum*, *O. lupi* and *Thelazia* spp.). The multiple sequence alignment viewer tool (recently implemented as function in the BLAST interface) was used to identify the core sequence alignment and to retrieve all related information (accession number, name, voucher code, host source, and geographical origin). Both, *cox*1 and 12S rRNA sequence datasets, referred here as dataset A and B respectively, were mapped using accession number and voucher code to construct a multi-gene dataset (dataset C).

### Species delimitation and phylogenetic analysis

To assess the genetic diversity throughout A, B and C datasets, the all-against-all sequence comparison with genetic distance clustering was performed using the software TaxI2 Tool^56^. A *priori*-defined intraspecific genetic distance thresholds of 0.15 and 0.02 for the *cox*1 and 12S rRNA sequences, respectively, were assessed to identify representative haplotypes as previously described^13,47^. To validate the haplotypes delineation, reference sequences for each haplotype were selected from A, B and C datasets and aligned using MAFFT^57^. Sequences from dataset C were concatenated using Sea view^58^. The designation of a reference sequence for each haplotype was considered when the DNA sequence showed a complete query cover within the core sequence alignment or placed at the branch level within the EPA-ng placement. While putative haplotypes were defined when the sequences lack the complete query cover and placed at the leaf level of the trees by the EPA-ng placement.

From all sequence alignments, the ML phylogeny was performed with 1000 bootstrap replications within IQTREE software^59^. Using model finder (implemented as function in IQTREE), the TIM2 (+F+R4), TIM3 (+F+I+G4) and the GTR (+F+R5) models were selected to infer the *cox*1, 12S rRNA and concatenated trees, respectively on Galaxy Server^60^. The remaining query sequences, from each dataset, were aligned against the reference sequences using Hmmer v3.3.2 software^61^ and phylogenetically placed on the ML phylograms using the evolutionary placement algorithm of the EPA-ng v0.3.8 software^62^. For more accurate phylogenetic placement, the heuristic classification was deactivated using the no-heur flag^62^ and searched using iTOL v5 software^63^. A correct placement of sequences was considered when the like weight ratio was ≥ 0.85. For each tree, the results of haplotype delineation, sequence placement, informative site for the amino acid alignment, number of human, domestic and wild animal cases, were used to annotate each tree within iTOL v5 software^63^.

### Spatial distribution of haplotypes

The delineated haplotypes were geographically mapped using QGIS version 3.0.0^25^. Global administrative boundaries were retrieved from the GADM^64^. Specifically, two different maps were built; one on eye worms and one concerned with the remaining species. The Jenks algorithm (Natural Breaks) was used to represent the availability of sequence data per country. The final geographical plotting of each haplotype was performed using country centroids and the point displacement tool.

## Supplementary Information

Supplementary Table S1.

Supplementary Figure S1.

## Data Availability

All data are provided within the manuscript or as Supplemental Files. Descriptive pipeline and dataset used in the present study are available as a GitHub repository.

## Abbreviations

VBHs: Vector-borne helminths
ASAP: Assemble species by automatic partitioning
ABGD: Automatic barcode gap discovery
GMYC: Generalized mixed Yule-coalescent
PTP: Poisson tree processes
ML: Maximum likelihood
CoSA: Core sequence alignment
EPA-ng: Evolutionary placement algorithm-next generation
GADM: Database of global administrative areas
