# Supplementary material for: Genetic and geographical delineation of zoonotic vector-borne helminths of canids
Source: Sci Rep. 2022 Apr 24;12:6699. doi: 10.1038/s41598-022-10553-w (PMC9035454; doi:10.1038/s41598-022-10553-w)
Supplement: Supplementary file 2 — Supplementary Figure S1. [file 41598_2022_10553_MOESM2_ESM.pdf]

Genetic and geographical delineation of zoonotic vector-borne helminths of canids

Younes Laidoudi 1,2, Samia Bedjaoui 3, Maria Stefania Latrofa 1, Angela Fanelli 4, Filipe Dantas-Torres5, Domenico Otranto 1,6*

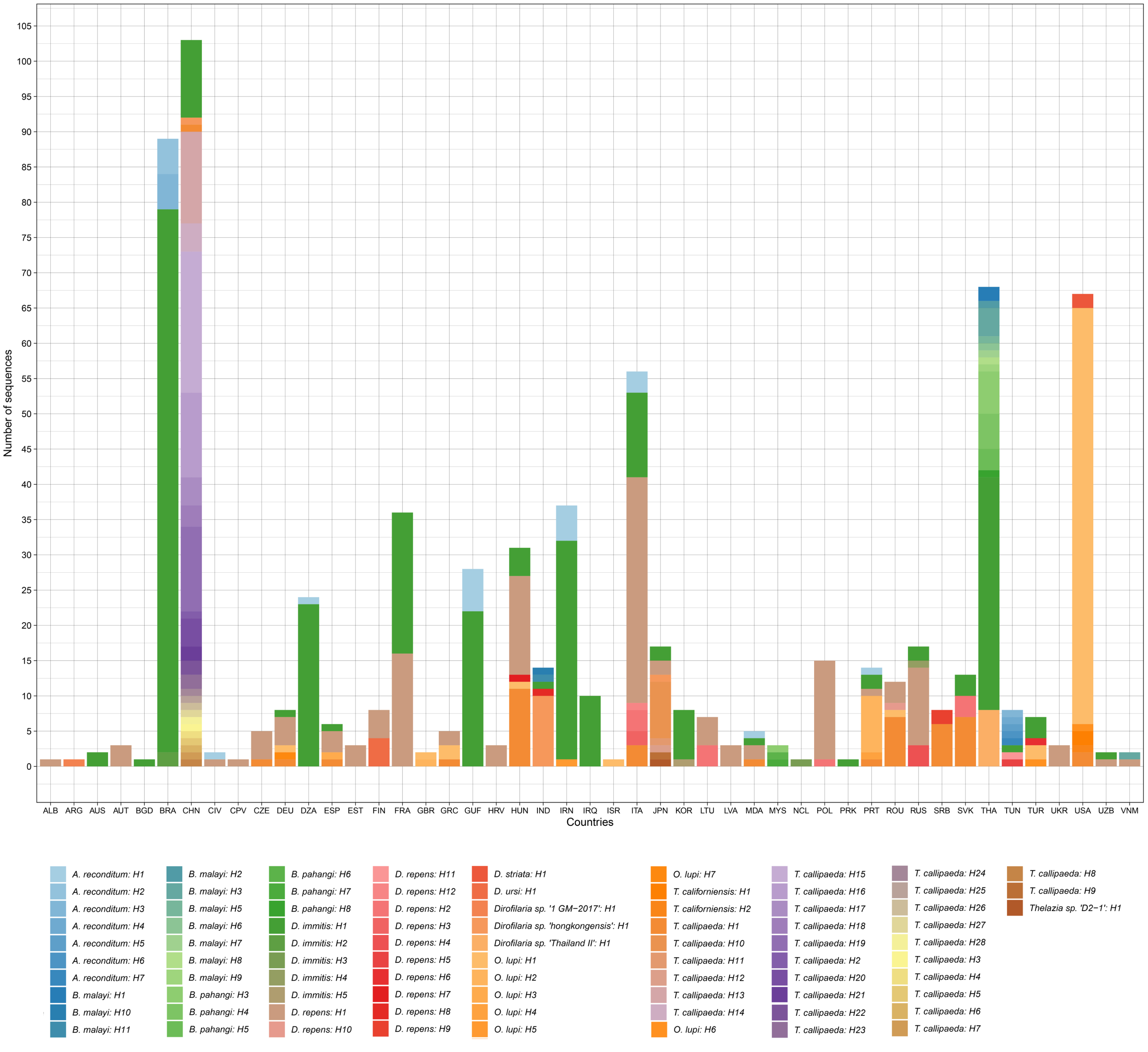

Supplemental file 2. Coloured bare chart showing the distribution of VBH haplotypes per countries. Number of DNA sequences available for each haplotype from each country were plotted using R software. Country codes are: ALB: Albania, DZA: Algeria, ARG: Argentina, AUS: Australia, AUT: Austria, BGD: Bangladesh, BRA: Brazil, CPV: Cape Verde, CHN: China (People's Rep. of), CIV: Cote D'Ivoire, HRV: Croatia, CZE: Czech Republic, EST: Estonia, FIN: Finland, FRA: France, GUF: French Guiana, DEU: Germany, GRC: Greece, HUN: Hungary, IND: India, IRN: Iran, IRQ: Iraq, ISR: Israel, ITA: Italy, JPN: Japan, PRK: Korea (Dem. People's Rep.), KOR: Korea (Rep. of), LVA: Latvia, LTU: Lithuania, MYS: Malaysia, MDA: Moldova, NCL: New Caledonia, POL: Poland, PRT: Portugal, ROU: Romania, RUS: Russia, SRB: Serbia, SVK: Slovakia, ESP: Spain, THA: Thailand, TUN: Tunisia, TUR: Turkey, UKR: Ukraine, GBR: United Kingdom, USA: United States of America, UZB: Uzbekistan, VNM: Vietnam
